# The West Texas Medical Society Defeats Sanitary Legislation

**Published:** 1903-04

**Authors:** 


### TEXAS MEDICAL JOURNAL.

AUSTIN, TEXAS.

A MONTHLY JOURNAL OF MEDICINE AND SURGERY.

EDITED AND PUBLISHED BY
F. E. DANIEL, M. D.

ASSOCIATE EDITORS:

WITTEN BOOTH RUSS, M. D.,
San Antonio, Texas.

P. M. PAYNE, M. D.,

Brownwood, Texas.

Published Monthly at Austin, Texas. Subscription price $1.00 a year in advance.

Eastern Representative: John Guy Monihan, St. Paul Building, 220 Broadway, New York City.

Official organ of the the West Texas Medical Association, the Houston District Medical Association, the Austin District Medical Society, the Brazos Valley Medical Association, the Galveston County Medical Society, and several others.

### THE WEST TEXAS MEDICAL SOCIETY DEFEATS SANITARY LEGISLATION.

*Et tu, Brute?*

The Legislature adjourned at noon April 1. It was next day reassembled in extra session upon call of the Governor. It is understood that no further efforts to secure legislation in the interest of the public health will be considered.

It is greatly to be deplored that the work of the State Medical Association's committee on State Board of Health was not more successful. An elaborate and very comprehensive and complete bill had been prepared (See *Texas Medical Journal,* January, 1903), covering every phase of sanitation, and providing for registration of vital statistics. This bill was killed in its incipiency by failure of the members of the committee to agree, and was never introduced. Two members objected to making the State Health Officer ex-officio president of the board. One of them had, in conference with Drs. Daniel and Tabor, at the New Braunfels meeting of the West Texas Medical Association, in October, 1902, consented to this, and it was understood and agreed that the quarantine laws and the authority of the State Health Officer were to be let alone. Upon this agreement between Drs. Daniel, Paschal and Tabor, State Health Officer Tabor consented to become a member of the committee, and to co-operate with the State Medical Association to get the bill passed, and he was added to the committee by request of the chairman. When the bill was presented to the members of the committee Drs. Paschal and Blaylock declined to sign it. Thereupon Dr. Tabor withdrew his support, and declined to "have anything more to do with it." Thus was undone the work whereby the opposition of the Quarantine Department (heretofore always fatal to the State Association's efforts) was removed and a cordial co-operation was assured. The chairman sent in his resignation, and turned the matter over to the remaining four members. No action was taken by them after the withdrawal of Drs. Daniel and Tabor, and Dr. Paschal advised to let the matter go by default and "report back to the Association (after the adjournment of the Legislature) that the committee could not agree." My resignation was not accepted.

Under these circumstances, I felt that rather than abandon all efforts and report back to the Association, "nothing done," it was our duty to try to secure legislation in the interest of the public health, even if we never got a board. Hence the second bill. Realizing the impracticability of getting a board, I felt that the majority of our committee should use the discretion the situation called for. Is it specifically a *board of health* they want, and a board of health or *nothing*? as advocated by Dr. Paschal, or is it sensible, salutary and effective sanitary legislation for the protection of the lives of the people? If the latter, what matter as to the means, if the end be reached? Dr. Tabor and I had abundantly satisfied ourselves that a State Board of Health was impossible. That is one reason why Dr. Tabor declined to join me and Drs. Coleman and Saunders in putting in the Board of Health bill as the action of the majority, and why we sought to *do something* by a less objectionable method.

Senators Davidson of DeWitt and Savage are two of the strongest men in the Senate. Senator Davidson said: "No effort to get a State Board of Health will succeed, as the conditions in this State require autocratic power in the matters of epidemics and quarantine."

Senator Savage said: "I can't say what other members of the Public Health Committee will do, but speaking for myself I shall oppose it." There was a pronounced sentiment against it.

Seeing that no effort would be made by the other members of the committee, Drs. Daniel and Tabor drew up a comprehensive bill, which provided for (See *Texas Medical Journal* for February):

A Bureau of Vital Statistics.

County and City Boards of Health.

An Assistant State Health Officer.

A Sanitary Code (under which measures of prevention could be enforced against other diseases than those now guarded against by quarantine).

A Chemist, as provided for by the law now inoperative.

Railway Sanitation, and a provision to make effective certain laws now dead because of lack of penalties to enforce them.

Thus it was sought to perfect the health machinery of the State, and make possible effective sanitation and preventive medicine. Our laws take cognizance of nothing save quarantinable diseases.

This bill would have accomplished the ends it had been hoped to secure by a State Board of Health. It was approved by Drs. Coleman and Saunders of the committee, who signed it, together with Drs. Daniel and Tabor. This bill was introduced in the Senate by Senator Harper of Limestone County, at the request of the chairman of the State Association's committee, and was duly reported favorably by the Public Health Committee (of which our venerable Dr. A. M. Douglas, Senator from Hill, was chairman), and on Friday, March 6, it was "pending business," and special order for 3 o'cock p. m. The indications were most favorable that it would pass without opposition or material change.

Just then the unexpected happened; the most surprising interference by the West Texas Medical Society! Drs. Paschal, Bell and Burleson (the latter two eye specialists), a committee from that society, arrived in Austin at 3 p. m. on the afternoon when the bill was to be called up. Without the knowledge of Dr. Daniel, the chairman of the committee (of which Dr. Paschal is a member), or of Dr. Tabor (also a member), and without so much as doing either of us the courtesy to let us know of their presence or their intentions, these gentlemen went into the Senate and had their Senator, Hicks, to suppress the bill! That is, it was "recommitted (to the grave, in which so many former efforts have been laid)." These gentlemen then went before the Senate committee on Public Health and protested against the passage of the bill on the ground that it did not provide for a State Board of Health, in accordance with the instructions of the State Medical Association! They repudiated the authority of the majority of the committee to act for the Association, and said it did not represent their wishes. They proposed to let the whole matter go over until another meeting of the Legislature—two years hence. This killed the bill—the second attempt to *do* something.

I should state that three days later (March 9) Dr. Paschal wrote me that he would withdraw his objections to the Board of Health bill if we would substitute it for the one he had protested against and killed. It was too late. The Senate committee declined to even consider the proposition.

After so long Drs. Daniel and Tabor succeeded in resurrecting a small fragment of the wrecked bill—that providing for a registration of vital statistics, and it was, near the close of the session, reported favorably, and put through the Senate and House, and was signed six minutes before those bodies adjourned. It was not discovered that it had been emasculated in committee room until after it became a law. That committee knocked out the clause providing for a Registrar, defining his duties, and fixing his salary; knocked out the appropriation. Thus the bill will be inoperative, unless the Legislature, now in called session, sees fit to include in the appropriation bill a sum sufficient to make the bill operative. Interested readers will find on page 334 of the February number of this Journal the part stricken out (Sec. 2). The Governor should, and probably will, veto it, as it is inoperative.

Elsewhere will be found this bill in full. Also Dr. Johnson's Railway Sanitation bill, now a law. It is the only legislation secured that is worth a cent.

